# Two sides of the same coin: distinct neuroanatomical patterns predict crystallized and fluid intelligence in adults

**DOI:** 10.3389/fnins.2023.1199106

**Published:** 2023-05-25

**Authors:** Hui Xu, Cheng Xu, Zhenliang Yang, Guanghui Bai, Bo Yin

**Affiliations:** ^1^Department of Neurosurgery, The Second Affiliated Hospital and Yuying Children’s Hospital of Wenzhou Medical University, Wenzhou, Zhejiang, China; ^2^Peter Boris Centre for Addictions Research, St. Joseph’s Healthcare Hamilton, McMaster University, Hamilton, ON, Canada; ^3^School of Psychology and Cognitive Science, East China Normal University, Shanghai, China; ^4^Faculty of Psychology, Tianjin Normal University, Tianjin, China; ^5^Department of Radiology, The Second Affiliated Hospital and Yuying Children’s Hospital of Wenzhou Medical University, Wenzhou, Zhejiang, China

**Keywords:** crystallized intelligence, fluid intelligence, neuroanatomy, morphometry, machine learning, elastic net regression

## Abstract

**Background:**

Crystallized intelligence (Gc) and fluid intelligence (Gf) are regarded as distinct intelligence components that statistically correlate with each other. However, the distinct neuroanatomical signatures of Gc and Gf in adults remain contentious.

**Methods:**

Machine learning cross-validated elastic net regression models were performed on the Human Connectome Project Young Adult dataset (*N* = 1089) to characterize the neuroanatomical patterns of structural magnetic resonance imaging variables that are associated with Gc and Gf. The observed relationships were further examined by linear mixed-effects models. Finally, intraclass correlations were computed to examine the similarity of the neuroanatomical correlates between Gc and Gf.

**Results:**

The results revealed distinct multi-region neuroanatomical patterns predicted Gc and Gf, respectively, which were robust in a held-out test set (*R*^2^ = 2.40, 1.97%, respectively). The relationship of these regions with Gc and Gf was further supported by the univariate linear mixed effects models. Besides that, Gc and Gf displayed poor neuroanatomical similarity.

**Conclusion:**

These findings provided evidence that distinct machine learning-derived neuroanatomical patterns could predict Gc and Gf in healthy adults, highlighting differential neuroanatomical signatures of different aspects of intelligence.

## 1. Introduction

General intelligence is defined as a general capability to understand complex ideas, adapt flexibly to the changing environment, solve problems, and engage in critical reasoning (Neisser et al., 1996; Gottfredson, 1997). Markers of neural substrates in brain regions and genetic biomarkers have been closely linked to intelligence (Posthuma et al., 2002; Genç et al., 2018), prompting the use of neuroimaging techniques to uncover the neural signature of intelligence. Furthermore, general intelligence has been postulated to consist of two independent components, crystallized intelligence (Gc) and fluid intelligence (Gf) (Cattell, 1943). While Gc reflects our ability to acquire skills through knowledge and experience and is related to verbal ability and general knowledge (Deary et al., 2007; Yuan et al., 2018), Gf refers to the capacity for problem-solving and logical reasoning and is suggested as one of the most important features associated with various cognitive abilities (Varriale et al., 2018). Despite the evidence that Gc and Gf are regarded as distinct intelligence components that statistically correlate with each other (Cattell, 1943; Li et al., 2004), it remains contentious whether there are distinct neuroanatomical signatures of Gc and Gf in adults.

An increasing number of functional magnetic resonance imaging (MRI) studies have found that Gf is linked with multiple cortical regions, which is postulated by the Parieto-Frontal Integration Theory (P-FIT) (Gray et al., 2003; Jung and Haier, 2007; Cipolotti et al., 2022). Based on P-FIT, Gf is linked to the executive network, which includes the dorsolateral prefrontal cortex, inferior and superior parietal lobules, and anterior cingulate gyrus (Jung and Haier, 2007). This explains the goal-directed behavior that is expressed by individual differences in Gf (Barbey et al., 2013; Barbey, 2018). Additionally, substantial evidence from structural MRI (sMRI) studies found higher Gc expression, which remained stable over time. The Gc level was associated with greater gray matter volume (GMV) reduction, and thinning of the cortex thickness (CT) (Yuan et al., 2018). Moreover, individual differences in Gc may depend on declarative knowledge stored in the temporal lobe and inferior prefrontal cortex, leading to widespread cortical region differences across individuals (Martin and Chao, 2001; McClelland and Rogers, 2003; Gainotti, 2006). Furthermore, Gf and Gc exhibit distinct trajectories of development (McArdle et al., 2000). However, these studies investigated the neural substrates of Gc and Gf using different models of modalities. These studies had small sample sizes for brain-intelligence associations using MRI (Marek et al., 2022), which led to low sensitivity for true effects (i.e., type I error) and increased risk for false positives (i.e., type II error) (Button et al., 2013).

Recently, studies started adopting larger samples to characterize the neuroanatomical correlates of Gc and Gf. One study used a large cohort of adults from the Human Connectome Project (HCP) and reported higher performance in Gf, which was associated with cortical expansion in regions related to working memory, attention, and visuospatial processing. In contrast, Gc was associated with thinner CT and higher cortical surface area (CSA) in language-related networks (*N* = 740) (Tadayon et al., 2020). Another Adolescent Brain Cognitive Development study (*N* = 10,652) conducted a double generalized linear model to assess the independent association between the mean and dispersion of CT/CSA and intelligence. It was found that higher intelligence in preadolescents was associated with higher mean CT in orbitofrontal and primary sensory cortices but with lower CT in the dorsolateral and medial prefrontal cortex, particularly in the rostral anterior cingulate (Zhao et al., 2022). However, these two studies were conducted using mass univariate approaches without cross-validation (CV), which might increase the risks of overfitting. In contrast, machine learning approaches with CV can assess and prevent overfitting more effectively than univariate approaches, ultimately leading to more generalized findings. One example of a machine learning approach is elastic net regression (ENR), which is an ideal approach to analyzing a large number of inter-correlated variables or predictors (Zou and Hastie, 2005; Owens et al., 2022). One study tested numerous machine learning algorithms for their effectiveness in the context of neuroimaging data and found that ENR models with CV performed well over a range of sample sizes as compared to other approaches (Jollans et al., 2019).

Several recent machine learning studies with CV comprehensively investigated predictive intelligence. Two moderately large studies (*N* = 415 and 392, respectively) found that distinct functional and structural connections contributed to the prediction of individual Gc and Gf (Dhamala et al., 2021), and the findings revealed neurobiological features of the functional connectome of Gc and Gf across the sexes (Dhamala et al., 2022). Additionally, another study (*N* = 308) reported that absolute GMV enabled significant predictions of individual intelligence scores (Hilger et al., 2020). However, these studies had several limitations. Firstly, the samples were enrolled from datasets with a relatively small sample size (less than 500). Secondly, these studies only investigated functional and structural connections or one neuroanatomical measure, GMV, and their relationship with intelligence. However, cortical GMV comprises CT and cortical surface area (CSA), which are known to be distinct morphological features of the cortical architecture (Tadayon et al., 2020). Both CT and CSA have distinct developmental trajectories and uncorrelated genetic backgrounds (Storsve et al., 2014), suggesting that CSA and CT should be considered separate morphometric features in neurodevelopment (Panizzon et al., 2009; Xu et al., 2023b).

To address these limitations, the current study used a machine learning approach to predict Gc and Gf from CSA, CT, and GMV. Data were drawn from the HCP, which remains one of the largest studies to date with contemporaneously collected Gc, Gf, and sMRI data. This study conducted ENR models with the CV approach, which is well suited to assess the overfitting and generalization of findings (Xu et al., 2023a). This approach simultaneously investigates all brain morphological variables as predictors of a target. Hence, this approach elucidates the neuroanatomical structures that are uniquely important to Gc and Gf. As a secondary strategy, this study also used a traditional univariate approach (linear mixed effects modeling) to confirm the presence of a univariate relationship between Gc and Gf and the neuroanatomical features contributing to the ENR models. Furthermore, intraclass correlation analyses were performed to examine the neuroanatomical pattern similarity of Gc and Gf. This study aimed to investigate whether Gc and Gf could be effectively predicted in an independent sample using a machine learning approach and uncover the distinct neuroanatomical patterns of Gc and Gf in adults.

## 2. Materials and methods

### 2.1. Participants

In this study, the HCP release S1200 dataset was used. Participants were recruited at Washington University in St. Louis over 2 days between August 2012 and October 2015 (Van Essen et al., 2012). The protocols were approved by each institution’s research ethics board. All participants provided written informed consent in accordance with the Declaration of Helsinki. All participants were young adults between 22 and 35 years old. The exclusion criteria were as follows: history of psychiatric disorder, substance abuse, neurodevelopmental disorder or damage, cardiovascular disease, severe health conditions (such as diabetes, multiple sclerosis, cerebral palsy, premature birth), or MRI contraindications (large tattoos, non-removable piercings, metal devices in the body or claustrophobia, etc.). The complete details of the inclusion and exclusion criteria and the informed consent for participants can be found in references (Van Essen et al., 2012, 2013). Some participants were excluded from further analysis due to the following reasons: missing sMRI scans, missing demographic data, and missing behavioral data. A total of 1,089 participants (90.75% of the initial sample size) were included in the final analysis (Table 1).

**TABLE 1:** Demographic characteristics of sample (*N* = 1089).

Metric	M (SD) or percent
Age	28.83 (3.68)
**Sex**	
Female	54.27%
Male	45.73%
**Total family income**	
<$10,000	7.16%
10K−19,999	7.99%
20K−29,999	12.49%
30K−39,999	12.03%
40K−49,999	10.38%
50K−74,999	20.75%
75K−99,999	13.50%
≥ 1,00,000	15.70%
**Education level**	
≤ 11 years	3.49%
12 years	13.77%
13 years	6.34%
14 years	12.40%
15 years	6.06%
16 years	42.15%
≥ 17 years	15.79%

M, mean; SD, standard deviation. These demographic variables were used as covariates in the following model analyses.

### 2.2. Intelligence assessment

**FIGURE 1:**
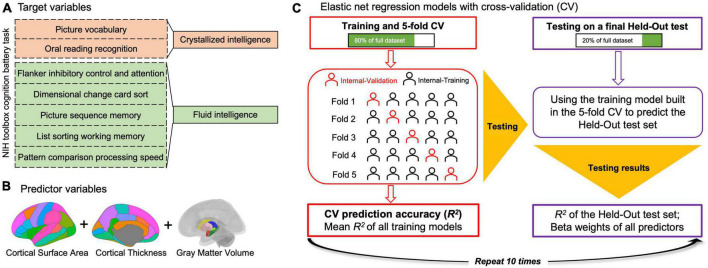
Schematic of elastic net regression (ENR) model analyses conducted. ENR models were built in which crystallized intelligence or fluid intelligence was the target, respectively, **(A)** and the predictors were regional sMRI variables (i.e., the cortical thickness and cortical surface area of each cortical region, gray matter volume of each subcortical region, and total intracranial volume) **(B)**. A modified coefficient of determination (*R*^2^) was calculated as the measure of prediction accuracy for each model. All ENR analyses were repeated 10 times to ensure stability of findings across different train/test splits and results across repetitions were averaged **(C)**.

Cognitive ability was assessed by the NIH Toolbox Cognition Battery with extensively validated neuropsychological tasks (Mungas et al., 2014). Two composite scores (crystallized cognition composite and fluid cognition composite) were derived from the scores of participants when performing NIH Toolbox Cognitive Battery tasks (Mungas et al., 2014). Gc was measured by Picture Vocabulary and Oral Reading Recognition Tests, which assessed language and verbal skills. Likewise, Gf was measured using the Dimensional Change Card Sort, Flanker Inhibitory Control and Attention Test, Picture Sequence Memory, List Sorting Working Memory, and the Pattern Comparison Processing Speed Test, which broadly assessed processing speed, memory, and executive functioning (Figure 1A).

### 2.3. MRI data acquisition and pre-processing

In the HCP dataset, T1-weighted structural images were collected using a 32-channel head coin on a 3T Siemens Skyra scanner (Siemens AG, Erlanger, Germany) with the following scanning parameters: isotropic resolution = 0.7 mm^3^, field of view = 224 mm × 240 mm, matrix size = 320 × 320, repetition time = 2,400 ms, echo time = 2.14 ms, inversion time = 1,000 ms, flip angle = 8°, and 256 sagittal slices. Data were reconstructed and pre-processed using a modified version of the FreeSurfer pipeline (Fischl et al., 2004) in FreeSurfer Image Analysis Suite version 5.3^1^ (Fischl, 2012). For details of acquisition parameters, reconstruction, and pre-processing of the HCP sMRI data, see references (Van Essen et al., 2012; Glasser et al., 2013) and Supplementary materials. All structural images were reviewed by a technician immediately after acquisition to ensure scans were without any significant problems (i.e., artifacts and substantial movement). For a detailed explanation of HCP quality control, check reference (Marcus et al., 2013). The quantitative measures of CT and CSA for cortical regions were defined by the Desikan atlas (Desikan et al., 2006), while the GMV for subcortical regions from the ASEG parcellation and intracranial volume (ICV) was derived in FreeSurfer (Fischl, 2012).

### 2.4. Data analyses

ENR model analyses (Figure 1) were conducted in Python using Scikit-Learn (Pedregosa et al., 2011) and the Brain Predictability toolbox (Hahn et al., 2021). LME model analyses were performed using R (Version 4.1.3^2^) and RStudio (“Ghost Orchid” Release; see text footnote 2), with the lme4 package (Version 1.1-28) (Bates et al., 2015).

#### 2.4.1. Elastic net regression model analyses

To remove the covariance (e.g., demographic variables, including age, sex, total family income, and education level), residual covariates were removed from a pool of variables, comprising Gc and Gf. The ENR models were built with Gc or Gf as the dependent variable. Hence, the predictors (i.e., independent variables) of the model-building algorithm were regional sMRI variables (i.e., the CT and CSA of each cortical region, the GMV of each subcortical region, and total ICV; Figure 1B). The model aimed to investigate neuroanatomical patterns that could predict Gc and Gf.

A modified coefficient of determination (*R*^2^) was calculated as a measure of accuracy for each model. All elastic net analyses were repeated 10 times to validate findings across different train/test splits, and the results across multiple repetitions were averaged (Figure 1C).

Initially, 20% of the total participants were selected as the held-out test set. For the remaining participants, a 5-fold CV was used to build and test five separate elastic net regression models. In this approach, the training data were split into five equal groups (i.e., “folds”). A model was then built using four of the 5-folds (i.e., the training data) and tested on the 5-fold (i.e., the validation set) to determine its accuracy. After five repetitions, with each fold serving as the test set exactly once, the mean of the five models was used to predict the held-out test set.

Within this 5-fold CV, hyperparameter tuning was performed in the training set with a nested 3-fold CV. A random hyperparameter search algorithm was used on 200 randomly selected combinations of hyperparameters (Alibrahim and Ludwig, 2021). In the 3-fold CV, the training data were split into 3-folds in each of the five model-building phases. Within each of the 3-folds, 200 randomly selected combinations of parameters were tested, and the best combination was selected. The combination that yielded the best accuracy from all the folds was used to build a model for 5-fold iteration in the outer loop.

#### 2.4.2. Linear mixed effects model analyses

To better interpret elastic net regression analyses, a secondary analysis was conducted to test the association of Gc and Gf with each sMRI variable from the final elastic net regression model. The linear mixed effects (LME) model analyzed each sMRI variable as a fixed effect. Demographic variables (sex, age, education level, and total family income) and ICV were fixed effects, and family ID was used as a random effect. The Gc or Gf was the dependent variable. The *P* < 0.05 after Bonferroni correction was used to indicate significance. Regions included in ERN models were only considered as neural correlates of Gc and Gf if they were also associated in the same direction in the univariate analyses.

#### 2.4.3. Intraclass correlation analyses

To examine the neuroanatomical distinctiveness of Gc and Gf, absolute similarity coefficients (i.e., intraclass correlation (ICC)) were calculated across the entire set of sMRI features. The regression coefficients for each regional brain measure from LME models and beta weights from ENR models served as the elements of ICC analyses. The double-entry intraclass correlations (McCrae, 2008), which accounted for absolute similarities in magnitude and direction of the neuroanatomical profiles of Gc and Gf, were used to quantify the degree of absolute neuroanatomical similarity between Gc and Gf. These indices were computed separately across CSA features, CT features, and GMV features, and across all sMRI features again. The neuroanatomical similarity between Gc and Gf was interpreted using cut-offs based on “poor reliability/replicability” (ICC = 0.00−0.50), “moderate reliability/replicability” (ICC = 0.50−0.75), “good reliability/replicability” (ICC = 0.75−0.90), and “excellent reliability/replicability” (ICC = 0.90−1.00) (Koo and Li, 2016). Additionally, Pearson correlations were conducted in sMRI features between Gc and Gf accordingly.

## 3. Results

### 3.1. ENR models

**FIGURE 2:**
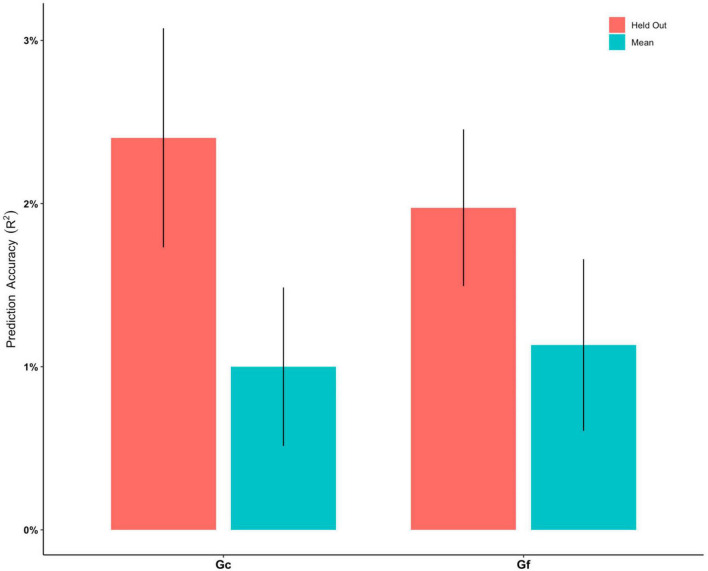
Prediction accuracy (*R*^2^) for elastic net regression models to predict crystalized intelligence (Gc) and fluid intelligence (Gf), respectively. “Mean” indicate the mean *R*^2^ of all models built in the training phase. “Held Out” indicates the all *R*^2^ of all models from the training phase being tested on the held-out test set. Error bars stand for standard error of mean.

**FIGURE 3:**
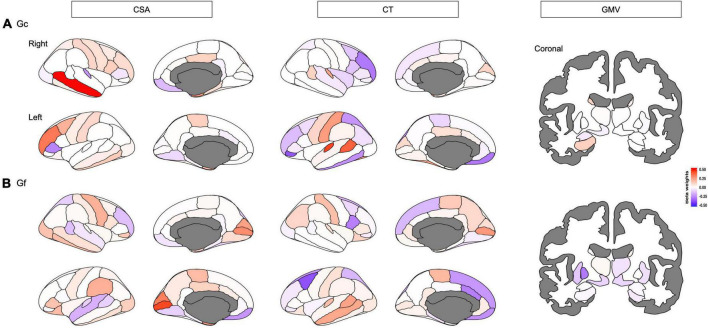
Distinct neuroanatomical patterns were indexed by beta weights of different features including cortical surface area (CSA), and cortical thickness (CT) of each cortical region, and gray matter volume (GMV) of each subcortical that predicted **(A)** crystalized intelligence (Gc) and **(B)** fluid intelligence (Gf), respectively. Red-shaded brain regions or bars indicate positive beta weights, while blue-shaded brain regions or bars indicate negative beta weights.

The ENR model predicted Gc with *R*^2^ of 1.00% after a 5-fold cross-validation. The *R*^2^ was 2.40% when predicting the held-out test set across 10 repetitions of the ENR procedure (Figure 2 and Supplementary Table 1). The pattern of regions that contributed to the mode (beta weights ranging between −0.3744 and 0.5356) included CSA and CT of the widespread frontal, parietal, and temporal regions (e.g., rostral middle frontal gyrus, medial orbitofrontal cortex, caudal middle frontal, posterior cingulate cortex, and caudal anterior cingulate cortex), and GMV of the subcortical regions, including the bilateral hippocampus and left thalamus (Figure 3A). Brain regions with positive/negative beta weights in the ENR model are reported in Supplementary Table 2.

The ENR model predicted Gf with an *R*^2^ of 1.13% after a 5-fold cross-validation. The *R*^2^ was 1.97% when predicting the held-out test set across 10 repetitions of the ENR procedure (Figure 2 and Supplementary Table 1). The pattern of regions that contributed to the mode (beta weights ranging between −0.3866 and 0.4309) included CSA and CT of the widespread frontal, parietal, and temporal regions (e.g., rostral middle frontal gyrus, medial orbitofrontal cortex, caudal middle frontal, superior and inferior parietal lobule, posterior cingulate cortex, and caudal anterior cingulate cortex), and GMV of the subcortical regions, including the bilateral nucleus accumbens and left pallidum (Figure 3B). Brain regions with positive/negative beta weights in the ENR model are reported in Supplementary Table 3.

### 3.2. LME models

**FIGURE 4:**
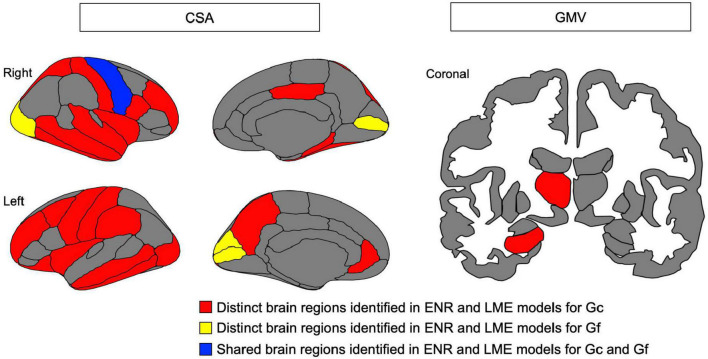
Brain map for cortical surface area (CSA) of cortical region and gray matter volume (GMV) of subcortical region identified as contributing to predict crystalized intelligence (Gc) and (B) fluid intelligence (Gf), respectively, in elastic net regression (ENR) models and found to be significantly associations in linear mixed effect (LME) models. Red indicates distinct brain regions identified in ENR and LME models for Gc; yellow indicates distinct brain regions identified in ENR and LME models for Gf; blue indicted shared brain regions identified in ENR and LME models for both Gc and Gf.

Linear mixed effects model analyses (Table 2) revealed the association between Gc and the CSA of widespread cortical regions (including bilateral rostral middle frontal gyrus, caudal middle frontal, superior frontal gyrus, and parahippocampal gyrus) and the GMV of subcortical regions (including the bilateral hippocampus and left thalamus). However, Gf was significantly associated with only CSA in limited cortical regions (including bilateral the pericalcarine fissure). According to the ENR model, the brain regions that were significant in the LME model for both Gc and Gf are displayed in Figure 4.

**TABLE 2:** Significant sMRI correlates of Gc and Gf in linear mixed effect models after Bonferroni correction.

Hemisphere	Region	*B*	SE	*t*	*P*_*Bonferroni*_	*R* ^2^
**Gc**						
**CSA**						
Right	Middle temporal gyrus	0.0066	0.0011	6.2413	0.0000	0.0428
Left	Rostral middle frontal gyrus	0.0033	0.0006	5.3011	0.0000	0.0342
Right	Rostral middle frontal gyrus	0.0030	0.0006	5.0896	0.0001	0.0330
Left	Lateral orbitofrontal cortex	0.0076	0.0016	4.8867	0.0002	0.0330
Left	Middle temporal gyrus	0.0058	0.0011	5.0853	0.0001	0.0328
Right	Precentral gyrus	0.0041	0.0009	4.7772	0.0003	0.0316
Left	Inferior temporal gyrus	0.0046	0.0009	5.0032	0.0001	0.0310
Left	Postcentral gyrus	0.0045	0.0009	4.8172	0.0003	0.0308
Right	Superior temporal gyrus	0.0052	0.0011	4.6094	0.0007	0.0304
Right	Postcentral gyrus	0.0043	0.0009	4.5031	0.0012	0.0284
Left	Precentral gyrus	0.0038	0.0009	4.3840	0.0020	0.0283
Left	Caudal middle frontal	0.0046	0.0010	4.4200	0.0017	0.0281
Right	Insula	0.0070	0.0016	4.2863	0.0031	0.0278
Right	Pars opercularis	0.0074	0.0017	4.4289	0.0017	0.0273
Right	Superior frontal gyrus	0.0023	0.0006	4.0546	0.0084	0.0269
Left	Superior temporal gyrus	0.0042	0.0011	4.0035	0.0104	0.0266
Left	Precuneus	0.0039	0.0010	3.9497	0.0130	0.0261
Left	Insula	0.0066	0.0016	4.0335	0.0092	0.0258
Left	Superior frontal gyrus	0.0021	0.0005	3.8975	0.0161	0.0258
Right	Fusiform gyrus	0.0040	0.0010	3.9316	0.0140	0.0252
Left	Lateral occipital gyrus	0.0029	0.0008	3.8073	0.0232	0.0251
Left	Rostral anterior cingulate	0.0106	0.0026	3.9977	0.0107	0.0249
Right	Superior parietal lobule	0.0027	0.0007	3.7923	0.0246	0.0247
Left	Supramarginal gyrus	0.0030	0.0008	3.7491	0.0292	0.0240
Right	Posterior cingulate	0.0076	0.0020	3.8591	0.0189	0.0240
Right	Parahippocampal gyrus	0.0151	0.0041	3.6537	0.0424	0.0235
Right	Frontal pole	0.0332	0.0088	3.7840	0.0256	0.0233
Right	Inferior temporal gyrus	0.0035	0.0010	3.6820	0.0380	0.0229
**GMV**						
	ICV	0.0000	0.0000	5.4403	0.0000	0.0380
Right	Hippocampus	0.0044	0.0011	4.0211	0.0097	0.0265
Left	Thalamus	0.0020	0.0005	3.9643	0.0123	0.0258
Left	Hippocampus	0.0039	0.0010	4.0502	0.0086	0.0251
**Gf**						
**CSA**						
Left	Pericalcarine fissure	0.0097	0.0023	4.3083	0.0028	0.0198
Right	Precentral gyrus	0.0041	0.0011	3.8765	0.0175	0.0164
Right	Lateral occipital gyrus	0.0035	0.0009	3.9168	0.0149	0.0159
Left	Cuneus	0.0102	0.0027	3.8300	0.0212	0.0158
Right	Pericalcarine fissure	0.0077	0.0021	3.6214	0.0479	0.0144

Gc, crystalized intelligence; Gf, fluid intelligence; sMRI, structural magnetic resonance imaging; B, unstandardized regression coefficient; SE, standard error; CT, cortical thickness; CSA: cortical thickness; GMV, gray matter volume. FDR, false discovery rate; *P*_Bonferroni_, *P*-value after Bonferroni correction; ICV, intracranial volume.

### 3.3. Neuroanatomical pattern similarity

intraclass correlation analyses of regression coefficients indicated that all sMRI features had poor similarity with all ICC below 0.50 between Gc and Gf (ICC = 0.1649−0.4761; Pearson’s r = 0.2614−0.5184; Table 3). Similarly, beta weights of all sMRI features reported poor similarity with all ICC below 0.40 between Gc and Gf (ICC = 0.0817−0.3910; Pearson’s r = 0.0812−0.4851; Table 3).

**TABLE 3:** Neuroanatomical similarity between Gc and Gf in different sMRI features.

Neuroanatomical similarity	Regression coefficients of LME models		Beta weights of ENR models	
	***r***	**ICC**	***r***	**ICC**
CSA features	0.4827	0.4761	0.0812	0.0817
CT features	0.5184	0.4579	0.3906	0.3910
GMV features	0.2614	0.1649	0.4851	0.2853
All sMRI features	0.4660	0.4388	0.2703	0.2712

Gc, crystalized intelligence; Gf, fluid intelligence; sMRI, structural magnetic resonance imaging. *r* represents Pearson’s *r* for the 156 sMRI features [68 indices of regional cortical surface area (CSA) + 68 indices of regional cortical thickness (CT) + 20 indices of gray matter volume (GMV) separately] for Gc and Gf; *ICC* represent intraclass correlation (ICC) between these same regions for Gc and Gf.

## 4. Discussion

This study aimed to provide a comprehensive examination of distinct neuroanatomical patterns to predict Gc and Gf in healthy adults using a cross-validated machine learning approach. Results of this approach indicted that distinct multi-region neuroanatomical patterns predicted Gc and Gf, respectively, with robust prediction accuracy in a held-out test set (*R*^2^ = 2.40% for Gc and *R*^2^ = 1.97% for Gf). Univariate LME model analyses supported the results, where the same brain regions identified in ENR models were significantly associated with Gc and Gf. Additionally, ICC findings exhibited poor neuroanatomical pattern similarity between Gc and Gf, indicating distinct neuroanatomical patterns to predict Gc and Gf. Taken together, these findings provided evidence that machine learning-derived distinct neuroanatomical patterns could predict Gc and Gf in healthy adults.

Interestingly, the ENR model indicated that Gc was more predictable than Gf from multi-region neuroanatomical patterns. Previous research proposed that Gc and Gf exhibited distinct developments and transformations across the lifespan (Cattell, 1967). While Gc is the ability to use previously learned knowledge and life experience, which are thought to be influenced by education and cultural factors, Gf is regarded as the ability to solve new problems using logical reasoning and adapt to unknown situations, which are thought to be more dependent on biological processes (Heaton et al., 2014). In this study, Gc reflected the scores of tasks such as vocabulary and decoding, while Gf reflected the scores of cognitive tasks including cognitive flexibility, working memory, and information processing speed (Mungas et al., 2014). The eloquent nature of the mapping between neuroanatomical morphometry profile and language, including vocabulary and reading as measured by Gc, may explain the higher variance of the scores when compared to Gc, which relies on brain functional networks for different cognitive functions. Another possible explanation for the higher predictability of Gc relative to Gf could be the impact of environment on neuroanatomical morphometry (Maggioni et al., 2020). Additionally, Gc is more stable throughout life and generally less susceptible to factors that affect cognitive function (e.g., mood and stress) (Riedel et al., 2002; O’Neill et al., 2020), resulting in the higher predictability of Gc over Gf. Additionally, a previous study found that cortical grey matter morphology provided little information about Gf and was probably incapable of predicting Gf (Oxtoby et al., 2019). This study validated this claim, whereby a low CSA led to low Gf predictivity. In this regard, Gf reflected higher cognitive functions, which were more dependent on large-scale brain networks (Gray et al., 2003; Barbey, 2018).

Moreover, the feature of neuroanatomical morphometry most linked to intelligence was CSA, which had more significant associations with Gc and Gf than CT or subcortical GMV. From the evolutionary perspective of the human cerebral cortex, the brain region is theorized to be enlarged mainly by the expansion of the surface area without a comparable increase in its thickness (Rakic et al., 2009). This suggests that the frontal and parietal surface area are enlarged first, followed by increasing thickness for young adults with higher intelligence. Evidence has verified that CSA and CT possess distinct genetic bases and developmental trajectories (Panizzon et al., 2009). Furthermore, gene expression is inversely correlated with development (Vidal-Pineiro et al., 2020). CSA and CT contribute to different aspects of intelligence (Gc and Gf). This study revealed the poor similarity between the neural correlates of Gc and Gf, evidenced by the low ICC in both ENR and LME models. The poor similarity between the neuroanatomical correlates of Gc and Gf supports the concept of distinct neuroanatomical patterns, suggesting that Gc and Gf may be “two sides of the same coin” (i.e., different aspects of intelligence have differential neuroanatomical signatures).

This study had several noteworthy strengths. This is a study for a machine learning-based approach to predict Gc and Gf using multiple metrics of the brain (i.e., CT, CSA, and GMV). The brain regions analyzed via the machine learning approach were largely supported by a univariate LME model, which validated the distinct brain regions to predict Gc and Gf. Additionally, the findings of this study were largely consistent with previous univariate analyses on the sMRI correlates of intelligence, highlighting the significance of the neuroanatomical correlates of intelligence. Furthermore, Gc and Gf were predicted by distinct neuroanatomical patterns with poor pattern similarity, which exhibited different neural substrates of distinct intelligence components in adults.

In retrospect, this study had several limitations. Firstly, the study used a cross-sectional design, which would discredit claims regarding the causality of the observed relationships. Future longitudinal studies should be performed to address this issue (Xu et al., 2018, 2019). Secondly, the current results are limited only to sMRI data, which could be further validated by resting-state functional MRI data or functional MRI data for related tasks (e.g., executive function) (Niu et al., 2020; Yang et al., 2020). Another direction to be explored is to determine whether the predictive model accuracy can be improved by an alternative machine learning approach. There is work suggesting that convolutional neural network modeling can outperform standard machine learning algorithms (Abrol et al., 2021). While this approach would require substantially more computational resources than the current analysis, this may improve the accuracy of predictive models.

## 5. Conclusion

In summary, using a cross-validated elastic net regression approach, this study indicated distinct neuroanatomical patterns that predicted Gc and Gf with robust accuracy in healthy adults. These findings verified the results of prior works to understand the neuroanatomical foundations of intelligence and demonstrate the utility of machine learning in this field of research. In addition, the distinct structural neural correlates of Gc and Gf were comprehensively studied and recognized for their involvement in different individual cognitive functions.

## Data availability statement

Publicly available datasets were analyzed in this study. This data can be found here: All data were provided by the Human Connectome Project, WU-Minn Consortium (Principal Investigators: David Van Essen and Kamil Ugurbil; 1U54MH091657) funded by the 16 NIH Institutes and Centers that support the NIH Blueprint for Neuroscience Research and the McDonnell Center for Systems Neuroscience at Washington University in St. Louis.

## Ethics statement

The studies involving human participants were reviewed and approved by the Second Affiliated Hospital and Yuying Children’s Hospital of Wenzhou Medical University. The patients/participants provided their written informed consent to participate in this study.

## Author contributions

HX: conceptualization, methodology, data curation, formal analysis, software, visualization, investigation, supervision, writing – original draft, and writing – review and editing. CX, ZY, and GB: methodology, and writing – reviewing and editing. BY: funding acquisition, project administration, supervision, and writing – review and editing. All authors contributed to the article and approved the submitted version.

## Footnotes

1
http://surfer.nmr.mgh.harvard.edu

2
https://www.r-project.org/
