# Supplementary material for: Two sides of the same coin: distinct neuroanatomical patterns predict crystallized and fluid intelligence in adults
Source: Front Neurosci. 2023 May 25;17:1199106. doi: 10.3389/fnins.2023.1199106 (PMC10249781; doi:10.3389/fnins.2023.1199106)
Supplement: Supplementary file 1 [file Data_Sheet_1.docx]

**Supplementary materials**

***MRI data analysis***

Anatomical MRI data were processed with FreeSurfer v7.3.2 (http://surfer.nmr.mgh.harvard.edu), which automatically creates a three-dimensional model of the cortical surface for morphometric measurements. Briefly, the cortical reconstruction includes motion correction and averaging of multiple volumetric T1 weighted images, removal of non-brain tissue with a hybrid watershed/surface deformation procedure, automated Talairach transformation, intensity normalization, tessellation of the gray and white matter boundary, automated topology correction. Then individual surfaces were inspected for segmentation errors between gray and white matter, and when needed manually fixed by a trained technician who was blinded to this study. Surface maps were smoothed with a full-width half-maximum Gaussian kernel of 30 mm and aligned across participants using a non-linear transformation in order to co-register the cortical folding patterns providing ~160000 surface vertex points where cortical thickness is automatically calculated. The vertices are co-located across all of the subjects to enable comparisons. Then, the average value of CT and CSA within 34 automatically cortical parcellations were defined by the Desikan atlas in each hemisphere (Desikan et al., 2006; Fischl et al., 2004). Finally, the values of these cortical surface indices for each cortical region were exported for analysis.

| **Table S1.** Prediction Accuracy (*R^2^*) for elastic net regression models to predict Gc and Gf respectively (Elastic net modeling was repeated 10 times, and these represent the average across 10 repetitions). | | | | |
| --- | --- | --- | --- | --- |
| Models |  | *R^2^* | | |
|  |  | Target: Gc |  | Target: Gf |
| Mean |  | 1.00% |  | 1.13% |
| Held Out |  | 2.40% |  | 1.97% |
| *Note.* “Mean” indicate the mean *R*^2^ of all models built in the training phase. “Held Out” indicates the mean R^2^ of all models from the training phase being tested on the held-out test set. Gf, fluid intelligence; Gc, crystalized intelligence. | | | | |

| **Table S2.** Beta weights for elastic net regression model: using sMRI variables to predict Gc in HCP dataset (sorted by beta weights). N.B., elastic net modeling was repeated 10 times, and these represent the average across 10 repetitions. | | | |
| --- | --- | --- | --- |
| Hemisphere | Region | Metric | Beta weights |
| Right | Middle temporal gyrus | CSA | 0.5356 |
| Left | Transverse temporal cortex | CT | 0.4338 |
| Left | Banks of the superior temporal sulcus | CT | 0.3909 |
| Left | Rostral middle frontal gyrus | CSA | 0.3405 |
| Right | Entorhinal cortex | CSA | 0.2608 |
| Left | Postcentral gyrus | CT | 0.2558 |
| Left | Fusiform gyrus | CT | 0.2354 |
| Right | Transverse temporal cortex | CT | 0.2295 |
| Right | Temporal pole | CT | 0.2255 |
| Left | Caudal middle frontal | CSA | 0.2047 |
| Left | Entorhinal cortex | CSA | 0.1640 |
| Left | Inferior temporal gyrus | CSA | 0.1347 |
| Right | Frontal pole | CSA | 0.1329 |
| Right | Pars opercularis | CSA | 0.1270 |
| Left | Hippocampus | GMV | 0.1220 |
| Left | Paracentral gyrus | CSA | 0.1215 |
| Right | Banks of the superior temporal sulcus | CT | 0.1153 |
| Right | Posterior cingulate | CSA | 0.1153 |
| Left | Precentral gyrus | CT | 0.1087 |
| Right | Cuneus | CT | 0.1078 |
| Right | Postcentral gyrus | CSA | 0.1069 |
| Left | Caudate | GMV | 0.1058 |
| Left | Postcentral gyrus | CSA | 0.1045 |
| Left | Cuneus | CT | 0.0990 |
| Right | Rostral middle frontal gyrus | CSA | 0.0924 |
| Right | Parahippocampal gyrus | CSA | 0.0869 |
| Left | Posterior cingulate | CT | 0.0824 |
| Right | Precentral gyrus | CSA | 0.0821 |
| Right | Entorhinal cortex | CT | 0.0623 |
| Right | Caudal middle frontal | CSA | 0.0525 |
| Right | Inferior temporal gyrus | CSA | 0.0478 |
| Left | Precentral gyrus | CSA | 0.0452 |
| Left | Middle temporal gyrus | CSA | 0.0443 |
| Right | Superior parietal lobule | CSA | 0.0443 |
| Right | Fusiform gyrus | CSA | 0.0441 |
| Right | Caudal anterior cingulate | CT | 0.0427 |
| Left | Caudal anterior cingulate | CT | 0.0365 |
| Left | Temporal pole | CSA | 0.0363 |
| Right | Caudate | GMV | 0.0361 |
| Left | Fusiform gyrus | CSA | 0.0354 |
| Left | Lingual gyrus | CT | 0.0350 |
| Left | Supramarginal gyrus | CT | 0.0348 |
| Left | Lateral orbitofrontal cortex | CSA | 0.0329 |
| Left | Superior temporal gyrus | CT | 0.0298 |
| Right | Posterior cingulate | CT | 0.0270 |
| Left | Lateral occipital gyrus | CSA | 0.0269 |
| Right | Fusiform gyrus | CT | 0.0230 |
| Left | Entorhinal cortex | CT | 0.0230 |
| Left | Parahippocampal gyrus | CT | 0.0228 |
| Left | Pericalcarine fissure | CT | 0.0214 |
| Right | Cuneus | CSA | 0.0173 |
| Right | Caudal anterior cingulate | CSA | 0.0160 |
| Left | Posterior cingulate | CSA | 0.0147 |
| Right | Lingual gyrus | CT | 0.0144 |
| Left | Temporal pole | CT | 0.0141 |
| Right | Postcentral gyrus | CT | 0.0136 |
| Left | Thalamus | GMV | 0.0124 |
| Left | Pericalcarine fissure | CSA | 0.0112 |
| Left | Rostral anterior cingulate | CSA | 0.0105 |
| Right | Precentral gyrus | CT | 0.0097 |
| Left | Pars orbitalis | CSA | 0.0092 |
| Right | Lateral orbitofrontal cortex | CSA | 0.0083 |
| Right | Rostral anterior cingulate | CSA | 0.0083 |
| Left | Precuneus | CSA | 0.0070 |
| Left | Medial orbitofrontal cortex | CSA | 0.0054 |
| Right | Lateral orbitofrontal cortex | CT | 0.0044 |
| Right | Isthmus of the cingulate | CT | 0.0043 |
| Right | Pars orbitalis | CSA | 0.0041 |
| Left | Insula | CT | 0.0039 |
| Left | Supramarginal gyrus | CSA | 0.0035 |
| Left | Inferior parietal lobule | CSA | 0.0030 |
| Left | Insula | CSA | 0.0018 |
| Right | Banks of the superior temporal sulcus | CSA | 0.0017 |
| Right | Supramarginal gyrus | CSA | 0.0014 |
|  | ICV | GMV | 0.0014 |
| Right | Lingual gyrus | CSA | 0.0009 |
| Right | Insula | CSA | 0.0008 |
| Right | Amygdala | GMV | 0.0002 |
| Right | Inferior temporal gyrus | CT | 0.0001 |
| Right | Inferior parietal lobule | CSA | -0.0003 |
| Right | Precuneus | CT | -0.0003 |
| Left | Isthmus of the cingulate | CT | -0.0009 |
| Left | Middle temporal gyrus | CT | -0.0012 |
| Right | Superior temporal gyrus | CSA | -0.0014 |
| Right | Inferior parietal lobule | CT | -0.0014 |
| Right | Ventral diencephalon | GMV | -0.0014 |
| Left | Transverse temporal cortex | CSA | -0.0020 |
| Right | Parahippocampal gyrus | CT | -0.0025 |
| Left | Lateral orbitofrontal cortex | CT | -0.0025 |
| Right | Paracentral gyrus | CSA | -0.0030 |
| Right | Precuneus | CSA | -0.0033 |
| Left | Pallidum | GMV | -0.0046 |
| Left | Pars opercularis | CSA | -0.0049 |
| Right | Pallidum | GMV | -0.0050 |
| Right | Middle temporal gyrus | CT | -0.0053 |
| Right | Pericalcarine fissure | CT | -0.0056 |
| Left | Precuneus | CT | -0.0060 |
| Left | Cerebellum cortex | GMV | -0.0065 |
| Right | Thalamus | GMV | -0.0078 |
| Left | Putamen | GMV | -0.0082 |
| Left | Accumbens area | GMV | -0.0092 |
| Left | Isthmus of the cingulate | CSA | -0.0096 |
| Right | Pericalcarine fissure | CSA | -0.0102 |
| Left | Frontal pole | CT | -0.0115 |
| Left | Pars opercularis | CT | -0.0130 |
| Left | Superior frontal gyrus | CT | -0.0137 |
| Right | Cerebellum cortex | GMV | -0.0143 |
| Right | Putamen | GMV | -0.0145 |
| Left | Cuneus | CSA | -0.0149 |
| Right | Medial orbitofrontal cortex | CT | -0.0154 |
|  | Brain stem | GMV | -0.0183 |
| Left | Frontal pole | CSA | -0.0193 |
| Right | Rostral anterior cingulate | CT | -0.0224 |
| Left | Parahippocampal gyrus | CSA | -0.0287 |
| Right | Isthmus of the cingulate | CSA | -0.0292 |
| Right | Lateral occipital gyrus | CSA | -0.0311 |
| Left | Ventral diencephalon | GMV | -0.0329 |
| Left | Rostral anterior cingulate | CT | -0.0353 |
| Right | Superior parietal lobule | CT | -0.0364 |
| Left | Caudal anterior cingulate | CSA | -0.0374 |
| Right | Pars orbitalis | CT | -0.0377 |
| Left | Pars triangularis | CT | -0.0401 |
| Left | Amygdala | GMV | -0.0404 |
| Right | Superior temporal gyrus | CT | -0.0452 |
| Right | Pars triangularis | CT | -0.0501 |
| Right | Superior frontal gyrus | CT | -0.0541 |
| Right | Pars triangularis | CSA | -0.0556 |
| Right | Frontal pole | CT | -0.0626 |
| Left | Inferior parietal lobule | CT | -0.0684 |
| Left | Caudal middle frontal | CT | -0.0749 |
| Left | Lingual gyrus | CSA | -0.0816 |
| Left | Paracentral gyrus | CT | -0.0889 |
| Right | Insula | CT | -0.0933 |
| Right | Caudal middle frontal | CT | -0.1244 |
| Right | Pars opercularis | CT | -0.1311 |
| Right | Accumbens area | GMV | -0.1412 |
| Left | Rostral middle frontal gyrus | CT | -0.1480 |
| Right | Medial orbitofrontal cortex | CSA | -0.1497 |
| Right | Temporal pole | CSA | -0.2151 |
| Right | Transverse temporal cortex | CSA | -0.2296 |
| Left | Superior parietal lobule | CT | -0.2343 |
| Left | Inferior temporal gyrus | CT | -0.2454 |
| Left | Pars triangularis | CSA | -0.2740 |
| Right | Rostral middle frontal gyrus | CT | -0.2994 |
| Left | Medial orbitofrontal cortex | CT | -0.3003 |
| Left | Pars orbitalis | CT | -0.3744 |
| *Note.* Gc: crystalized intelligence; sMRI: structural MRI; CT: cortical thickness of cortical region; CSA: cortical surface area of cortical region; GMV: gray matter volume of subcortical region, brain stem and intracranial volume (ICV). | | | |

| **Table S3.** Beta weights for elastic net regression model: using sMRI variables to predict Gf in HCP dataset (sorted by beta weights). N.B., elastic net modeling was repeated 10 times, and these represent the average across 10 repetitions. | | | |
| --- | --- | --- | --- |
| Hemisphere | Region | Metric | Beta weights |
| Left | Pericalcarine fissure | CSA | 0.4309 |
| Left | Cuneus | CSA | 0.3246 |
| Right | Pericalcarine fissure | CSA | 0.2925 |
| Right | Pericalcarine fissure | CT | 0.2659 |
| Left | Entorhinal cortex | CSA | 0.2509 |
| Right | Cuneus | CSA | 0.2435 |
| Left | Banks of the superior temporal sulcus | CT | 0.2390 |
| Left | Middle temporal gyrus | CT | 0.2272 |
| Left | Supramarginal gyrus | CSA | 0.2066 |
| Right | Precentral gyrus | CSA | 0.2047 |
| Left | Paracentral gyrus | CT | 0.1896 |
| Left | Paracentral gyrus | CSA | 0.1803 |
| Left | Lateral orbitofrontal cortex | CSA | 0.1757 |
| Left | Transverse temporal cortex | CT | 0.1702 |
| Right | Inferior temporal gyrus | CSA | 0.1695 |
| Right | Postcentral gyrus | CT | 0.1666 |
| Left | Pars triangularis | CSA | 0.1586 |
| Right | Lateral occipital gyrus | CSA | 0.1573 |
| Right | Pars orbitalis | CSA | 0.1433 |
| Right | Paracentral gyrus | CT | 0.1406 |
| Left | Lateral occipital gyrus | CSA | 0.1282 |
| Right | Entorhinal cortex | CSA | 0.1210 |
| Right | Lateral orbitofrontal cortex | CT | 0.1152 |
| Left | Posterior cingulate | CSA | 0.1127 |
| Left | Temporal pole | CSA | 0.1082 |
| Right | Inferior parietal lobule | CT | 0.1080 |
| Right | Precuneus | CT | 0.1016 |
| Right | Lingual gyrus | CSA | 0.0879 |
| Right | Transverse temporal cortex | CT | 0.0871 |
| Right | Cuneus | CT | 0.0837 |
| Left | Inferior temporal gyrus | CSA | 0.0822 |
| Left | Postcentral gyrus | CT | 0.0809 |
| Right | Middle temporal gyrus | CSA | 0.0807 |
| Left | Cerebellum cortex | GMV | 0.0769 |
| Right | Pars opercularis | CSA | 0.0765 |
|  | ICV | GMV | 0.0673 |
| Left | Lateral orbitofrontal cortex | CT | 0.0645 |
| Left | Lateral occipital gyrus | CT | 0.0604 |
| Right | Rostral anterior cingulate | CT | 0.0598 |
| Left | Pericalcarine fissure | CT | 0.0587 |
| Left | Precuneus | CSA | 0.0567 |
| Right | Cerebellum cortex | GMV | 0.0564 |
| Right | Postcentral gyrus | CSA | 0.0560 |
| Right | Pars triangularis | CT | 0.0537 |
| Left | Isthmus of the cingulate | CT | 0.0518 |
| Left | Precentral gyrus | CSA | 0.0518 |
| Left | Rostral anterior cingulate | CT | 0.0468 |
| Left | Caudal anterior cingulate | CSA | 0.0453 |
| Left | Inferior temporal gyrus | CT | 0.0383 |
| Right | Entorhinal cortex | CT | 0.0374 |
| Left | Temporal pole | CT | 0.0355 |
| Left | Lingual gyrus | CT | 0.0351 |
| Right | Caudate | GMV | 0.0346 |
| Right | Caudal middle frontal | CSA | 0.0342 |
| Right | Isthmus of the cingulate | CT | 0.0342 |
| Left | Supramarginal gyrus | CT | 0.0329 |
| Right | Caudal anterior cingulate | CSA | 0.0315 |
| Left | Superior parietal lobule | CSA | 0.0306 |
| Left | Inferior parietal lobule | CT | 0.0285 |
| Left | Superior temporal gyrus | CT | 0.0269 |
| Left | Insula | CSA | 0.0253 |
| Right | Posterior cingulate | CSA | 0.0249 |
| Right | Posterior cingulate | CT | 0.0247 |
| Left | Frontal pole | CSA | 0.0225 |
| Right | Medial orbitofrontal cortex | CT | 0.0224 |
| Right | Insula | CT | 0.0217 |
| Left | Precentral gyrus | CT | 0.0215 |
| Right | Insula | CSA | 0.0210 |
| Left | Thalamus | GMV | 0.0208 |
| Right | Paracentral gyrus | CSA | 0.0196 |
| Right | Supramarginal gyrus | CT | 0.0181 |
| Right | Supramarginal gyrus | CSA | 0.0179 |
| Right | Lateral orbitofrontal cortex | CSA | 0.0178 |
| Left | Superior frontal gyrus | CSA | 0.0147 |
| Right | Temporal pole | CSA | 0.0142 |
| Left | Middle temporal gyrus | CSA | 0.0130 |
| Left | Inferior parietal lobule | CSA | 0.0127 |
| Right | Inferior parietal lobule | CSA | 0.0120 |
| Left | Pars triangularis | CT | 0.0099 |
| Right | Transverse temporal cortex | CSA | 0.0097 |
| Right | Precuneus | CSA | 0.0097 |
| Right | Superior temporal gyrus | CT | 0.0096 |
| Right | Precentral gyrus | CT | 0.0080 |
| Right | Superior parietal lobule | CT | 0.0077 |
| Left | Pars opercularis | CT | 0.0064 |
| Left | Rostral middle frontal gyrus | CSA | 0.0039 |
| Left | Cuneus | CT | 0.0033 |
| Right | Parahippocampal gyrus | CT | 0.0032 |
| Left | Precuneus | CT | 0.0022 |
| Left | Caudate | GMV | 0.0017 |
| Right | Pallidum | GMV | 0.0003 |
| Right | Temporal pole | CT | 0.0001 |
| Left | Isthmus of the cingulate | CSA | -0.0009 |
| Right | Pars triangularis | CSA | -0.0017 |
| Right | Rostral anterior cingulate | CSA | -0.0031 |
| Left | Fusiform gyrus | CSA | -0.0035 |
| Right | Inferior temporal gyrus | CT | -0.0041 |
| Right | Middle temporal gyrus | CT | -0.0043 |
| Left | Caudal middle frontal | CSA | -0.0050 |
| Left | Postcentral gyrus | CSA | -0.0051 |
| Right | Superior frontal gyrus | CSA | -0.0060 |
| Left | Amygdala | GMV | -0.0060 |
| Right | Frontal pole | CT | -0.0068 |
| Right | Frontal pole | CSA | -0.0077 |
| Right | Caudal anterior cingulate | CT | -0.0085 |
| Left | Entorhinal cortex | CT | -0.0086 |
| Left | Posterior cingulate | CT | -0.0120 |
| Left | Pars opercularis | CSA | -0.0123 |
| Left | Fusiform gyrus | CT | -0.0131 |
| Right | Amygdala | GMV | -0.0181 |
| Left | Pars orbitalis | CSA | -0.0191 |
| Left | Hippocampus | GMV | -0.0199 |
| Right | Rostral middle frontal gyrus | CT | -0.0212 |
| Left | Rostral anterior cingulate | CSA | -0.0214 |
| Right | Isthmus of the cingulate | CSA | -0.0219 |
| Right | Banks of the superior temporal sulcus | CT | -0.0239 |
| Left | Insula | CT | -0.0279 |
| Right | Lateral occipital gyrus | CT | -0.0322 |
| Right | Parahippocampal gyrus | CSA | -0.0370 |
| Right | Pars orbitalis | CT | -0.0377 |
| Left | Parahippocampal gyrus | CSA | -0.0392 |
| Right | Lingual gyrus | CT | -0.0398 |
| Right | Hippocampus | GMV | -0.0400 |
| Left | Pars orbitalis | CT | -0.0465 |
| Left | Rostral middle frontal gyrus | CT | -0.0473 |
| Right | Superior temporal gyrus | CSA | -0.0548 |
| Right | Medial orbitofrontal cortex | CSA | -0.0584 |
| Right | Thalamus | GMV | -0.0605 |
| Left | Parahippocampal gyrus | CT | -0.0623 |
| Right | Putamen | GMV | -0.0722 |
| Right | Caudal middle frontal | CT | -0.0823 |
| Right | Fusiform gyrus | CT | -0.0839 |
| Right | Ventral diencephalon | GMV | -0.0854 |
| Right | Fusiform gyrus | CSA | -0.0893 |
| Left | Ventral diencephalon | GMV | -0.0932 |
| Left | Banks of the superior temporal sulcus | CSA | -0.1023 |
| Left | Lingual gyrus | CSA | -0.1024 |
| Left | Transverse temporal cortex | CSA | -0.1030 |
| Right | Superior parietal lobule | CSA | -0.1047 |
| Left | Putamen | GMV | -0.1206 |
| Right | Banks of the superior temporal sulcus | CSA | -0.1369 |
| Right | Rostral middle frontal gyrus | CSA | -0.1398 |
| Right | Superior frontal gyrus | CT | -0.1580 |
| Left | Superior parietal lobule | CT | -0.1600 |
| Left | Frontal pole | CT | -0.1603 |
| Left | Superior temporal gyrus | CSA | -0.1715 |
| Left | Superior frontal gyrus | CT | -0.2307 |
| Left | Medial orbitofrontal cortex | CSA | -0.2321 |
|  | Brain stem | GMV | -0.2383 |
| Left | Accumbens area | GMV | -0.2440 |
| Left | Medial orbitofrontal cortex | CT | -0.2462 |
| Right | Pars opercularis | CT | -0.2840 |
| Left | Caudal anterior cingulate | CT | -0.2845 |
| Left | Pallidum | GMV | -0.2943 |
| Right | Accumbens area | GMV | -0.3409 |
| Left | Caudal middle frontal | CT | -0.3866 |
| *Note.* Gf: fluid intelligence; sMRI: structural MRI; CT: cortical thickness of cortical region; CSA: cortical surface area of cortical region; GMV: gray matter volume of subcortical region, brain stem and intracranial volume (ICV). | | | |

**References**

Desikan, R. S., Ségonne, F., Fischl, B., Quinn, B. T., Dickerson, B. C., Blacker, D., Buckner, R. L., Dale, A. M., Maguire, R. P., Hyman, B. T., Albert, M. S., & Killiany, R. J. (2006). An automated labeling system for subdividing the human cerebral cortex on MRI scans into gyral based regions of interest. *Neuroimage*, *31*(3), 968-980. <https://doi.org/10.1016/j.neuroimage.2006.01.021>

Fischl, B., van der Kouwe, A., Destrieux, C., Halgren, E., Ségonne, F., Salat, D. H., Busa, E., Seidman, L. J., Goldstein, J., Kennedy, D., Caviness, V., Makris, N., Rosen, B., & Dale, A. M. (2004). Automatically parcellating the human cerebral cortex. *Cereb Cortex*, *14*(1), 11-22. <https://doi.org/10.1093/cercor/bhg087>
